# Acquired Lymphangiectasia of the Scrotum Successfully Treated with Radiofrequency Ablation: A Case Report with Dermoscopic Review

**DOI:** 10.1155/2023/7111912

**Published:** 2023-01-10

**Authors:** Dhan Keshar Khadka, Raksha Pathak, Sudha Agrawal, Sairil Pokharel

**Affiliations:** ^1^Department of Dermatology and Venereology, B. P. Koirala Institute of Health Sciences, Dharan, Nepal; ^2^Department of Dermatology and Venereology, Lumbini Provincial Hospital, Butwal, Nepal; ^3^Department of Pathology, B. P. Koirala Institute of Health Sciences, Dharan, Nepal

## Abstract

Lymphangiectasia is dilatation of normal superficial lymphatic vessels due to damage or obstruction of deep lymphatic vessels leading to increased lymphatic pressure and engorgement of dermal lymphatics due to varying causes. Lymphangiectasia clinically presents as thick-walled, translucent vesicles and papules with chronic lymphedema rarely involving the scrotum. Here we report a patient with acquired lymphangiectasia of the scrotum secondary to surgery for hydrocele successfully treated with radiofrequency ablation. We highlight the use of dermoscopy as a non-invasive diagnostic tool in lymphangiectasia.

## 1. Introduction

Lymphangiectasia is dilatation of normal cutaneous lymphatic channels due to the damage to previously normal lymphatic vessels leading to failure of drainage of lymph causing back pressure and dermal backflow [1]. Lymphangiectasias are also termed lymphangiectasis, acquired lymphangiomas, secondary lymphangiomas, and acquired lymphangioma circumscriptum. The suffix “-ectasia” means dilation or distension of a tubular structure and the suffix “-oma” means a tumour. Since there is distension of lymph vessels and not proliferation, “lymphangiectasia” is the appropriate term [2]. It occurs as a consequence of radiation, surgery, malignancy, infections like filariasis, tuberculosis, lymphogranuloma venereum, trauma, or pregnancy [3]. Lymphangiectasia clinically presents as thick-walled, translucent vesicles and papules with lymphedema often complicated with chronic discharge (oozing or sudden profuse), pain, itching, recurrent infections, and cosmetic disfigurement leading to physiological stress and affecting the quality of life [4–6]. Histologically, the dermis shows dilated and angular lymphatic vessels [7, 8]. Dilatation of deep dermal and subcutaneous lymphatic channels due to congenital malformation is termed as lymphangioma circumscriptum [8]. Clinical, histological, and dermoscopy features of lymphangiectasias and lymphangioma circumscriptum are similar [5, 9]. Here, we report a case of acquired lymphangiectasia of the scrotum along with dermoscopic and histopathological features and treatment outcome.

## 2. Case Report

**Figure 1:**
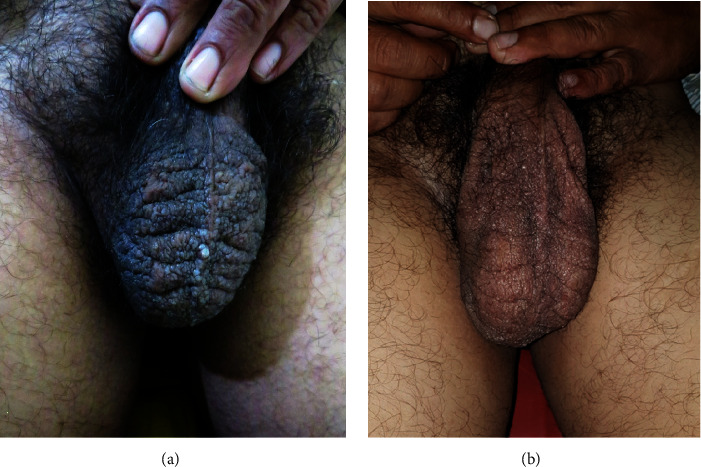
(a) Multiple whitish to skin-colored papules and vesicles on the scrotum with few nodules. (b) Clinical picture of the scrotum after 30 months of follow-up.

A 50 years male presented with a complaint of sudden frequent copious discharge of clear fluid from the scrotum for 5 years leading to wetting of undergarments . This caused discomfort and embarrassment affecting his daily activities and resulting in psychological stress. He also had a history of multiple whitish raised lesions on the scrotum for 7 years which increased in number gradually to cover most of the surface of the scrotum. He had undergone surgery for hydrocele 20 years ago. However, there was no history of trauma and infections at that site or past medical diseases. On examination, there were multiple, discrete to grouped, whitish to skin-colored papules and vesicles and a few nodules on the scrotum. Vesicles were clear fluid filled and few were hemorrhagic. The skin of the scrotum was thickened. The penis, testes, epididymis, and inguinal cord were normal (Figure 1(a)).

**Figure 2:**
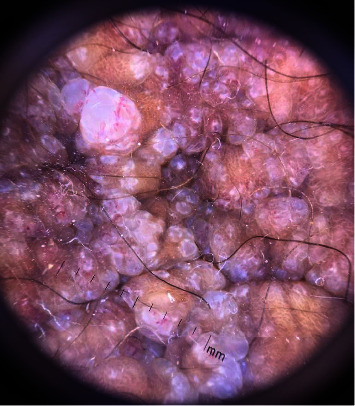
Dermoscopy shows multiple, densely distributed, round to oval, yellowish-white, reddish to purplish translucent lacunae surrounded by pale septa along with a few linear and punctate vessels and reddish to brownish scattered dots and globules.

Polarized dermoscopy of the scrotum (DermLite DL3 Nx10; 3Gen) revealed multiple, densely distributed, round to oval, yellowish-white, reddish to purplish translucent lacunae surrounded by pale septa along with few linear and punctate vessels and reddish to brownish scattered dots and globules (Figure 2).

**Figure 3:**
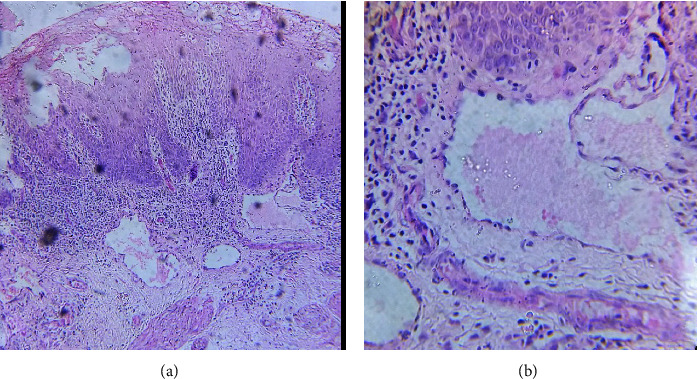
(a) Histopathological examination showing multiple dilated vessels on the upper dermis (H&E 10x). (b) Single layer of cells in the vessel wall and eosinophilic material in lumen (H&E 40x).

Ultrasound examination of the scrotum revealed a mild diffusely thickened scrotal wall. Histopathological examination of a representative lesion revealed multiple dilated vessels on the upper dermis with a single layer of cells and eosinophilic material in the lumen (Figures 3(a) and 3(b)). Based on the clinical, dermoscopic, and histological findings, the diagnosis of acquired lymphangiectasia of the scrotum was made.

The patient was treated with 4 sessions of radiofrequency ablation at 2–2.5 MHz (Megasurg Gold high-frequency radiosurgery unit, Dermaindia) under local anesthesia at 2-month intervals. The procedure was followed by supportive closed coconut dressing for 3 days with antibiotics and analgesics. The patient improved significantly following treatment. The number of lesions was reduced by 90%, discharge of fluid stopped completely, and the patient's quality of life improved considerably in 30 months of follow-up (Figure 1(b)).

## 3. Discussion

Lymphangiectasia is an acquired condition usually occurring between 40 and 60 years. It can develop anywhere on the body, common sites being upper limbs, chest, and axilla. Acquired lymphangiectasia of the scrotum is an uncommon manifestation. To the best of our knowledge, 21 cases have been reported till date mostly from Asian countries, and no case has been reported from Nepal (Table 1).

Literature suggests the age range from 9 to 65 years. There is history of trauma [3], surgery [3, 9–13], malignancy [3, 10, 11], radiation [3, 10], or infections like filariasis [10, 14, 15] and tuberculosis [16] few weeks to several years prior to appearance of skin lesions. Rarely etiology is unknown [6, 7]. 7 cases (35%) had a history of filariasis 1 month to 36 years prior to skin eruptions. In our case, there is history of surgery for hydrocele 13 years prior to skin lesions. Surgery can cause injury and obstruction of lymphatics leading to lymphangiectasia. However, we could not find the cause of hydrocele. Nepal being an endemic area for filariasis, it could be a possible etiology, but no history and documents suggestive for filariasis were available.

Histopathological examination of lymphangiectasia shows dilated and angular lymphatic vessels in the superficial and mid-dermis. In lymphangioma circumscriptum, lymphatic abnormalities often involve deep dermis and subcutis with smooth muscles in the walls of dilated lymphatics [8, 14]. The overlying epidermis shows varying degrees of hyperkeratosis, acanthosis, and papillomatosis. Dilated lymphatic vessels in the superficial and mid-dermis in our case were suggestive of lymphangiectasia. The immunohistochemistry tests help to differentiate between hemangiomas and lymphangiomas [8].

Few reports have explained the dermoscopic features of acquired lymphangiectasia with a single case involving the scrotum [15, 17, 18] (Table 2).

Therapy for lymphangiectasia is challenging because of the high recurrence rate, underlying lymphedema, risk for infections, and long healing time. Treatment options include complete surgical excision and grafting, cryotherapy, and CO_2_ laser vaporization [9]. The treatment modality used, outcome, and follow-up were mentioned in a few of the cases only. Surgical excision and grafting were done in one case with no recurrence for 19 months [10]. A case showed partial improvement with cryotherapy [3]. CO_2_ laser treatment was able to produce a good cosmetic result and significant improvement in quality of life in 2 cases [9, 14]. CO_2_ laser is a good treatment option as it is less destructive and easy to operate. The disadvantages of lasers are high cost and unavailability in resource-poor settings. None of the reports shows radiofrequency ablation as a treatment method. Our patient was successfully treated with four sessions of radiofrequency ablation with a good outcome in 30 months of follow-up. In case of unavailability of CO_2_ laser, radiofrequency ablation can be used as effective ablative therapy in lymphangiectasia of the scrotum.

## 4. Conclusion

The occurrence of acquired lymphangiectasia in the scrotum is rare. A detailed history should be taken to find out the cause. Dermoscopy is a tool that may give a diagnostic clue about the condition. We recommend radiofrequency ablation as a cost-effective method of treatment for acquired lymphangiectasia of the scrotum in resource-poor settings.

## Figures and Tables

**Table 1:** Reported cases of lymphangiectasia of the scrotum.

Case	Age at onset	Cause	Duration from cause to onset of lesions	Treatment and outcome	Reference
1	40 years	Rectum carcinoma followed by surgery, irradiation	4 years		[10] (case series)
2	40 years	Filariasis	10 years		
3	40 years	Lymphatic dysplasia	2 weeks		
4	55 years	Unknown	Unknown		
5	48 years	Filariasis	23 years		
6	50 years	Filariasis	14 years		
7	53 years	Filariasis	36 years		
8	47 years	Filariasis	22 years	Surgical excision and skin graft—no recurrence for 19 months	
9	34 years	Radical surgery for penile squamous-cell carcinoma	18 months		[11]
10	43 years	Bilateral varicose veins followed by sclerotherapy and ambulatory phlebectomy	20 years	CO_2_ laser ablation (6 sittings 1 month apart)—discharge stopped and no new lesions in 1-year follow-up	[9]
11	51 years	Filariasis	2 years	CO_2_ laser ablation (6 sittings in weekly interval)—lesions resolved	[17]
12	14 years	Scrofuloderma	2 years		[12]
13	54 years	Partial excision and eversion of sac for bilateral vaginal hydrocele	1 year		[13]
14	9 years	Orchiopexy of right testis for cryptorchidism	8 years		[14]
15	65 years	Unknown			[6]
16	17 years	Unknown			[7]
17	44 years	Squamous-cell carcinoma of the scrotum			[3] (case series)
18	33 years	Hodgkin's lymphoma, excision followed by radiotherapy	15 years		
19	23 years	Blunt injury to the genital region	11 years	Cryotherapy—partial improvement	
20	64 years	Adenocarcinoma			
21	59 years	Filariasis	1 month		[18]
Our case	43 years	Surgery for hydrocele	13 years	Radiofrequency ablation	

**Table 2:** Reported cases of lymphangiectasia with dermoscopic findings.

Cases	Sites	Cause	Dermoscopy findings	Histopathology correlation	References
1	Breast	Breast surgery	(i) Well-circumscribed, white-yellowish lacunae surrounded by pale septa (ii) Some lesions show scattered reddish areas and red lacunae	(i) Saccular dilations and ectatic lymphatic vessels lined by a single layer of endothelial cells in the dermis (ii) Inclusion of blood cells within lymphatic vessels	[15] (case series)
2	Breast	Breast surgery			
3	Breast	Breast surgery			
4	Vulva	Surgery/radiotherapy for carcinoma of the bladder	(i) Well-demarcated, round to oval red lacunae surrounded by white areas/lines (ii) Several punctiform and few irregular vessels		[16]
5	Scrotum	Filariasis	(i) Multiple skin-colored to erythematous nodules translucent pale blue lacunae (ii) Few radially arranged linear irregular vessels over the nodules	(i) Pale blue lacunae represents minimal inflammatory infiltrate and normal epidermal thickness (ii) Skin-colored or erythematous nodules represents acanthosis overlying inflammatory infiltrate which masks blue hue	[18]
Our case	Scrotum	Surgery for hydrocele	(i) Multiple, densely distributed, round to oval, yellowish-white, reddish to purplish translucent lacunae surrounded by pale septa (ii) Few linear and punctate vessels (iii) Reddish to brownish scattered dots and globules	(i) Dilated lymphatic vessels in the dermis (ii) Red to purplish color due to the inclusion of blood cells within lymphatic vessels	

## Data Availability

The data used to support the findings of this study are available from the corresponding author upon request.
